# Supplementary material for: Resting fMRI functional connectivity reflects fluctuations in inhibitory interneuron activity
Source: bioRxiv. 2026 Jun 28:2026.06.25.734567. Preprint. [Version 1] doi: 10.64898/2026.06.25.734567 (PMC13321047; doi:10.64898/2026.06.25.734567)
Supplement: 1 [file NIHPP2026.06.25.734567v1-supplement-1.pdf]

bioRxiv preprint doi: https://doi.org/10.64898/2026.06.25.734567; this version posted June 28, 2026. The copyright holder has placed this preprint (which was not certified by peer review) in the Public Domain. It is no longer restricted by copyright. Anyone can legally share, reuse, remix, or adapt this material for any purpose without crediting the original authors.

Supplemental Material for

Resting fMRI functional connectivity reflects fluctuations in inhibitory interneuron activity

Daniel Zaldivar[a,b,*], Lea Ives[a], Kenji W. Koyano[a,c], Rebecca Bhik-Ghanie[a], Brian E. Russ[a], Frank Q. Ye[a,b], David A. Leopold[a,b,d,*]

[a] Section on Cognitive Neurophysiology and Imaging, Systems Neurodevelopment Laboratory, National Institute of Mental Health, National Institutes of Health, Bethesda, MD 20892

[b] Neurophysiology Imaging Facility, National Institute of Mental Health, National Institute for Neurological Disorders and Stroke, National Eye Institute, National Institutes of Health, Bethesda, MD 20892

[c] Sector for Neuroplasticity and Learning Dynamics, Advanced Neuroimaging Center, National Institutes for Quantum Science and Technology, Chiba 263-8555 Japan

[d] National Institute for Neurological Disorders and Stroke, National Institutes of Health, Bethesda, MD 20892

bioRxiv preprint doi: https://doi.org/10.64898/2026.06.25.734567; this version posted June 28, 2026. The copyright holder has placed this preprint (which was not certified by peer review) in the Public Domain. It is no longer restricted by copyright. Anyone can legally share, reuse, remix, or adapt this material for any purpose without crediting the original authors.

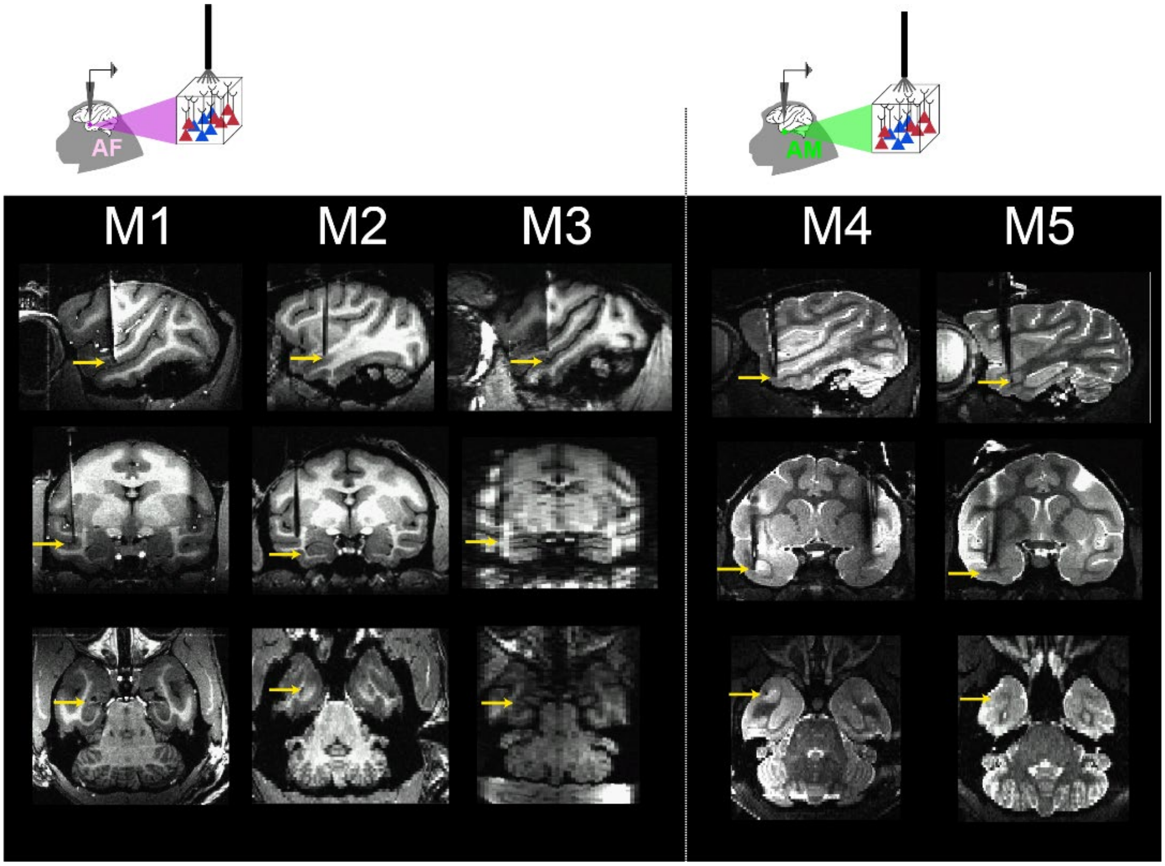

Figure S1 Targeting of functionally defined recording areas. Anatomical scans showing location of neurophysiological recordings in five subjects. Recordings were performed using chronically implanted 32 or 64 MR-compatible microwires. Subjects M1, M2, and M3 were implanted with electrodes in the anterior fundus face patch (left-side images) while M4 and M5 were implanted with electrodes in the anterior medial face patch.

bioRxiv preprint doi: https://doi.org/10.64898/2026.06.25.734567; this version posted June 28, 2026. The copyright holder has placed this preprint (which was not certified by peer review) in the Public Domain. It is no longer restricted by copyright. Anyone can legally share, reuse, remix, or adapt this material for any purpose without crediting the original authors.

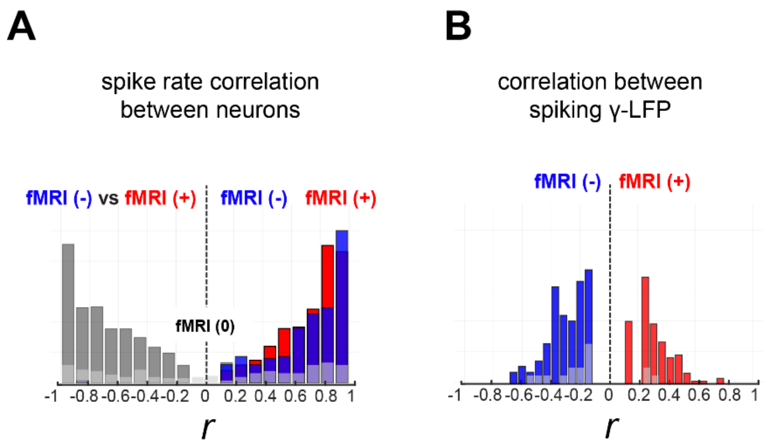

Figure S2. Interneuronal and spike-LFP correlations during resting state. (A) Distribution of spike-rate correlations between all simultaneously recorded neuron pairs. Pairwise correlations were computed either within each functional class (fMRI(+)/fMRI(+) in red; fMRI(–)/fMRI(–) in blue) or **across** classes (fMRI(+)/fMRI(–) in gray). Neuron pairs within the same class—both fMRI(+) and fMRI(–)—exhibit predominantly positive interneuronal correlations spanning a broad range of strengths, indicating coherent shared fluctuations within each population. In contrast, mixed fMRI(+)/fMRI(–) pairs cluster tightly around zero, consistent with a functional dissociation and reduced shared variability between the two classes. **(B)** Distribution of correlations between single-unit spiking activity and γ-band (40–100 Hz) LFP power. fMRI(–) neurons (blue) show strong negative correlations with γ-LFP power, whereas fMRI(+) neurons (red) exhibit positive correlations with γ-LFP power. This bivalent relationship suggests that the two neuronal classes participate in distinct local circuit states during rest, with fMRI(–) neurons preferentially associated with reductions in γ-band activity and fMRI(+) neurons associated with increases in γ-band activity.

bioRxiv preprint doi: https://doi.org/10.64898/2026.06.25.734567; this version posted June 28, 2026. The copyright holder has placed this preprint (which was not certified by peer review) in the Public Domain. It is no longer restricted by copyright. Anyone can legally share, reuse, remix, or adapt this material for any purpose without crediting the original authors.

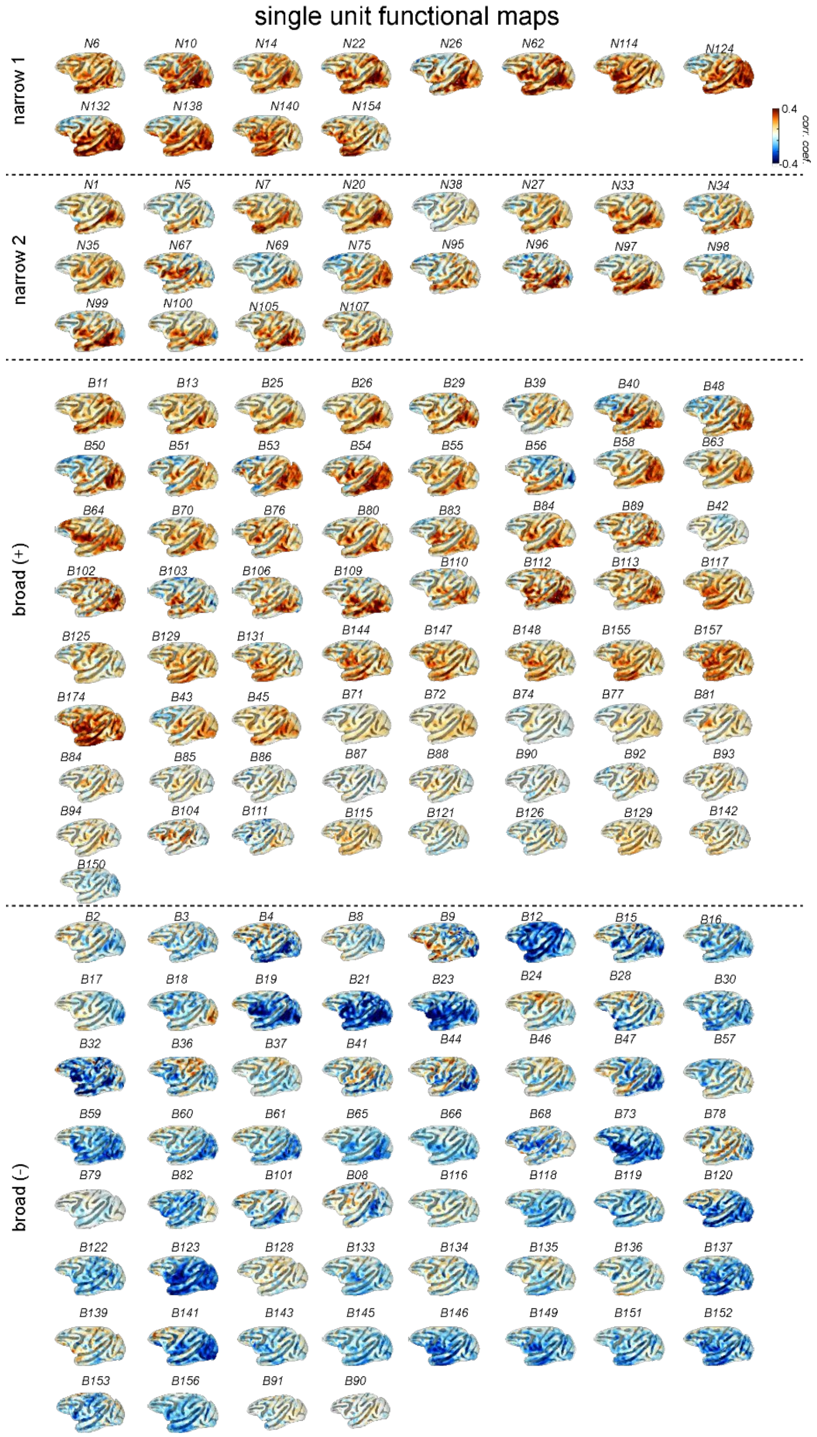

Figure S3 Single-unit functional maps from recorded signals in face patches. Each surface map represents single-unit functional map extracted from recordings across five monkeys. The maps are separated based on their cortical correlation map polarity and their cell type classification.

bioRxiv preprint doi: https://doi.org/10.64898/2026.06.25.734567; this version posted June 28, 2026. The copyright holder has placed this preprint (which was not certified by peer review) in the Public Domain. It is no longer restricted by copyright. Anyone can legally share, reuse, remix, or adapt this material for any purpose without crediting the original authors.

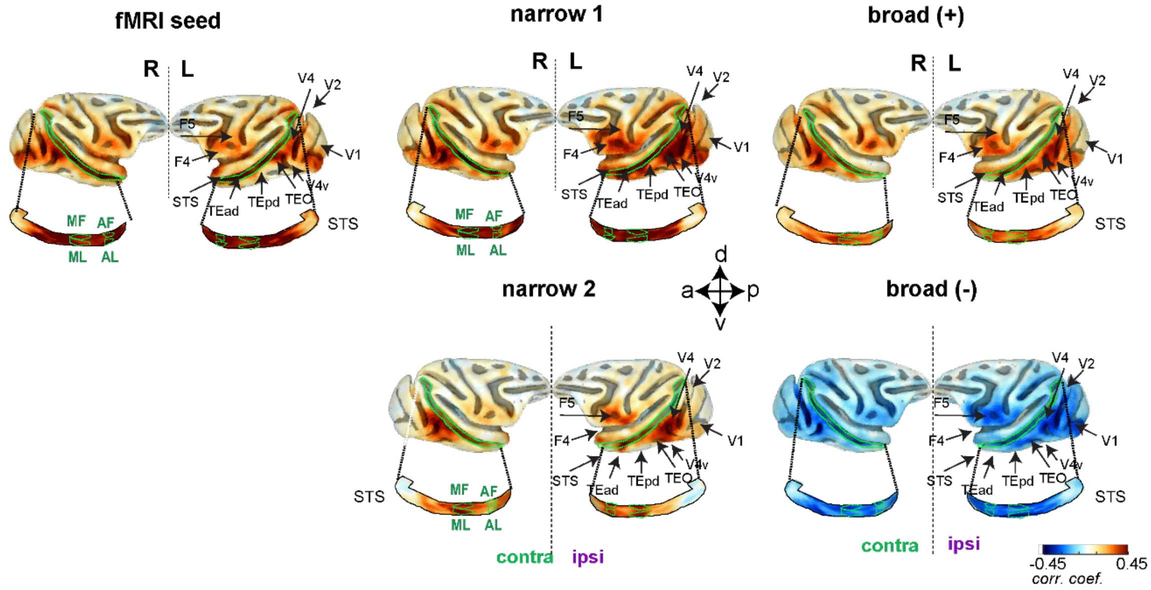

Figure S4 Bilateral functional connectivity for the different functional seeds. Lateral view of the macaque cortical surface showing functional connectivity (FC) for the fMRI seed and the four cell-types–defined groups. The Narrow groups (narrow 1 and narrow 2; top and bottom, respectively) consist exclusively of fMRI (+) neurons, whereas the Broad groups (broad (+) and broad (–); top and bottom, respectively) include both fMRI(+) and fMRI (–) neuron types. Boundaries of functionally defined face patches (green) are superimposed in the unfolded superior temporal sulcus (STS). Face-patch abbreviations: AL, anterior lateral; MF, middle fundus; ML, middle lateral.

bioRxiv preprint doi: https://doi.org/10.64898/2026.06.25.734567; this version posted June 28, 2026. The copyright holder has placed this preprint (which was not certified by peer review) in the Public Domain. It is no longer restricted by copyright. Anyone can legally share, reuse, remix, or adapt this material for any purpose without crediting the original authors.

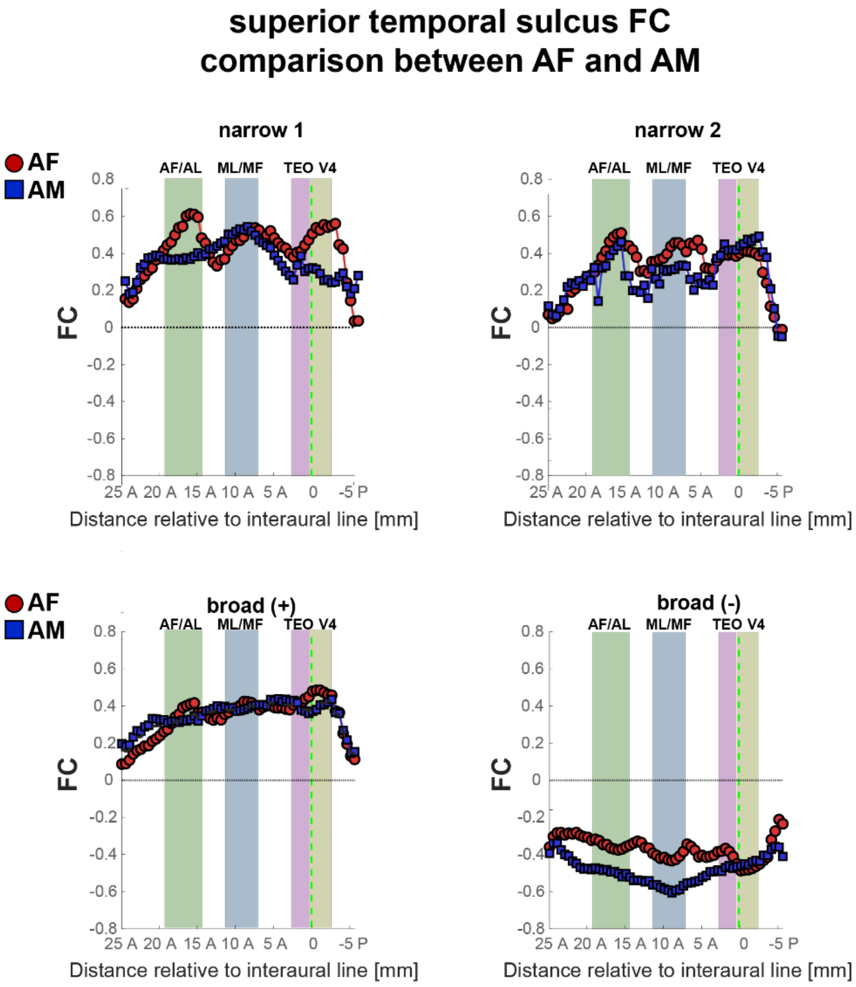

Figure S5 Superior temporal sulcus functional connectivity profiles for AF and AM recorded cells. Regional functional connectivity (FC) along the superior temporal sulcus (STS) is shown for cells extracted from AF face patches (red) and AM face patches (blue). The x-axis represents distance relative to the interaural line, spanning from posterior visual areas (V4) to more anterior temporal regions, including TEO, ML/MF, and AF/AL. Shaded bands indicate approximate anatomical boundaries of these regions. Data are shown separately for narrow face patches (narrow 1 and narrow 2; top row) and broad face patches with positive and negative connectivity profiles (broad (+) and broad (–); bottom row). Across conditions, AF and AM cells exhibit similar large-scale STS connectivity gradients, with systematic differences in magnitude and sign depending on patch type.

bioRxiv preprint doi: https://doi.org/10.64898/2026.06.25.734567; this version posted June 28, 2026. The copyright holder has placed this preprint (which was not certified by peer review) in the Public Domain. It is no longer restricted by copyright. Anyone can legally share, reuse, remix, or adapt this material for any purpose without crediting the original authors.

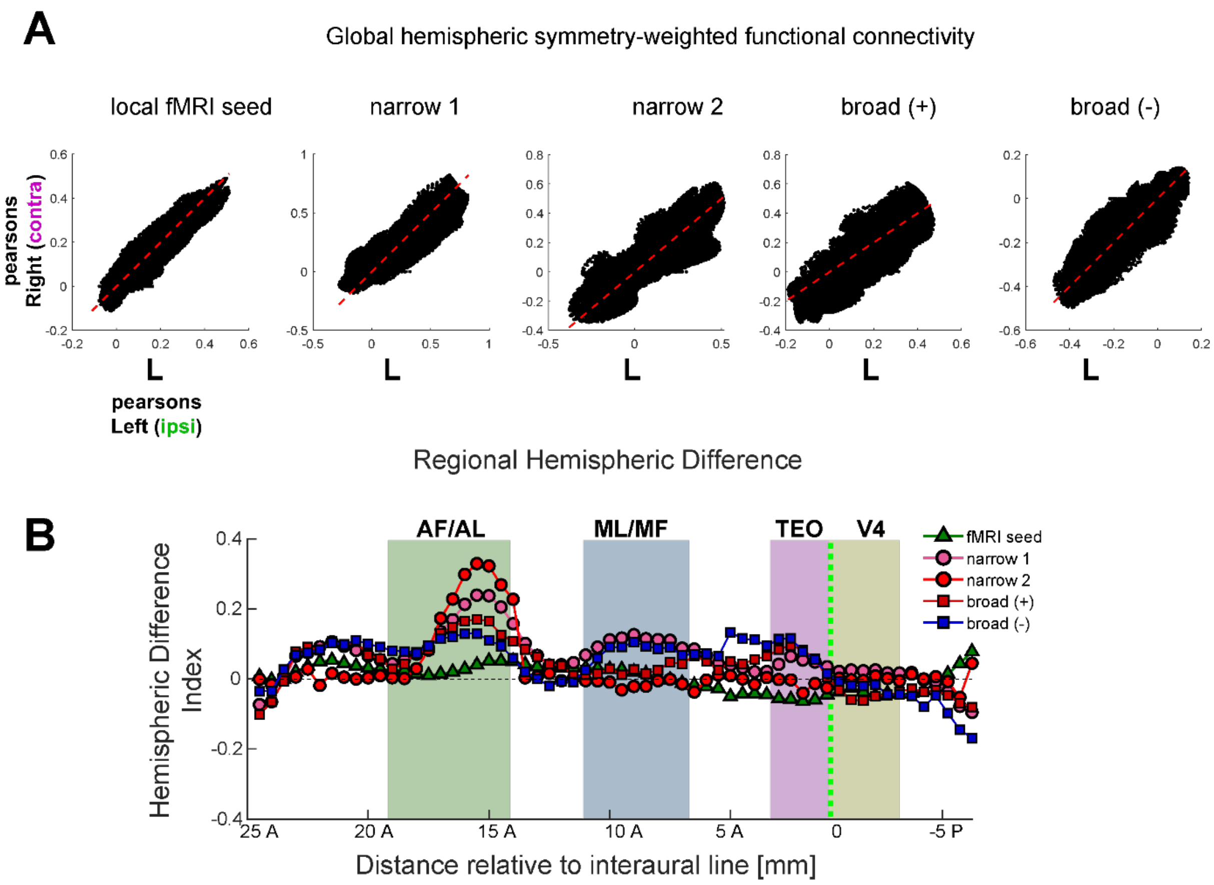

Figure S6. Global hemispheric symmetry of functional connectivity across neuron types. A. Interhemispheric correlations depict the overall connectivity patterns across different sets of functional seeds, including the local fMRI seed and seeds derived from distinct neuronal groups. The analysis shows the Pearson correlation of functional connectivity values between the left (ipsilateral) and right (contralateral) hemispheres for each neuronal group and the local fMRI seed. Each data point in the plots represents a spatially distributed voxel. The dashed red line indicates the unity line (L = R), highlighting the correspondence between hemispheres, while the blue line represents a weighted linear regression through the origin. The first panel displays correlations derived from the local fMRI seed, and the remaining panels show correlations for neuronal groups narrow 1 and narrow 2 (narrow-waveform neurons) and broad (+) and broad (–) (broad-waveform neurons). Across all groups, the functional connectivity demonstrates strong global symmetry between hemispheres. **B.** Regional hemispheric differences in functional connectivity along the STS axis. For each neuronal group and the local fMRI seed, we computed a hemispheric difference index (HDI) across the rostro-caudal axis of the superior temporal sulcus (STS). HDI was calculated as (L-R)/(L+R), where positive values indicate stronger connectivity in the left hemisphere (ipsilateral) and negative values indicate stronger connectivity in the right (contralateral). The resulting profiles show how lateralization varies regionally along the IT/V4 trajectory, revealing focal deviations from global symmetry that are specific to neuronal groups.

bioRxiv preprint doi: https://doi.org/10.64898/2026.06.25.734567; this version posted June 28, 2026. The copyright holder has placed this preprint (which was not certified by peer review) in the Public Domain. It is no longer restricted by copyright. Anyone can legally share, reuse, remix, or adapt this material for any purpose without crediting the original authors.

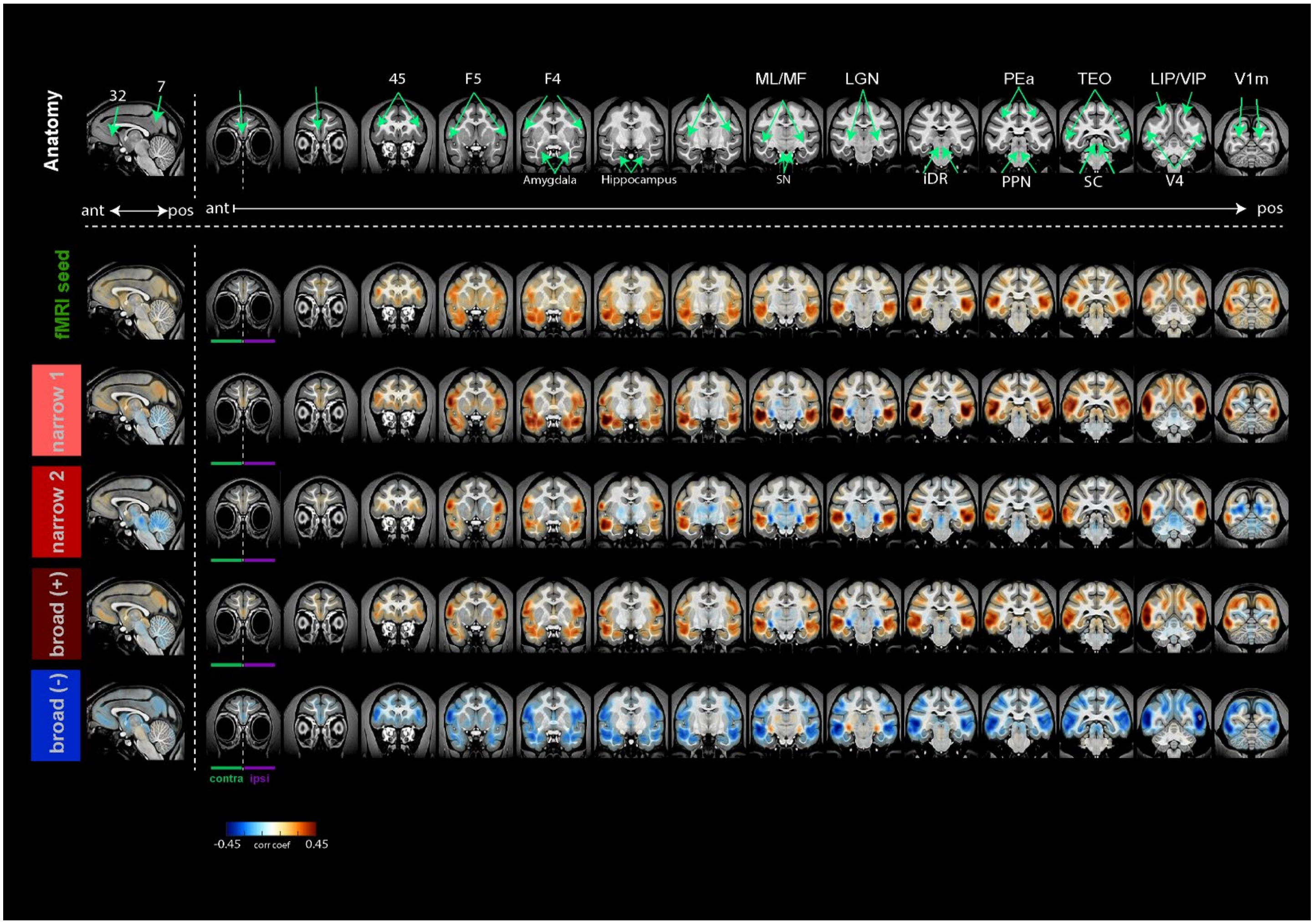

Figure S7 fMRI activation across groups of cells. Functional connetivity across the different groups of neurons. The top panel illustrates anatomical slices, with green arrows marking the anatomical locations of specific brain regions. The bottom panel depicts the functional connectivity across neuronal groups.

bioRxiv preprint doi: https://doi.org/10.64898/2026.06.25.734567; this version posted June 28, 2026. The copyright holder has placed this preprint (which was not certified by peer review) in the Public Domain. It is no longer restricted by copyright. Anyone can legally share, reuse, remix, or adapt this material for any purpose without crediting the original authors.

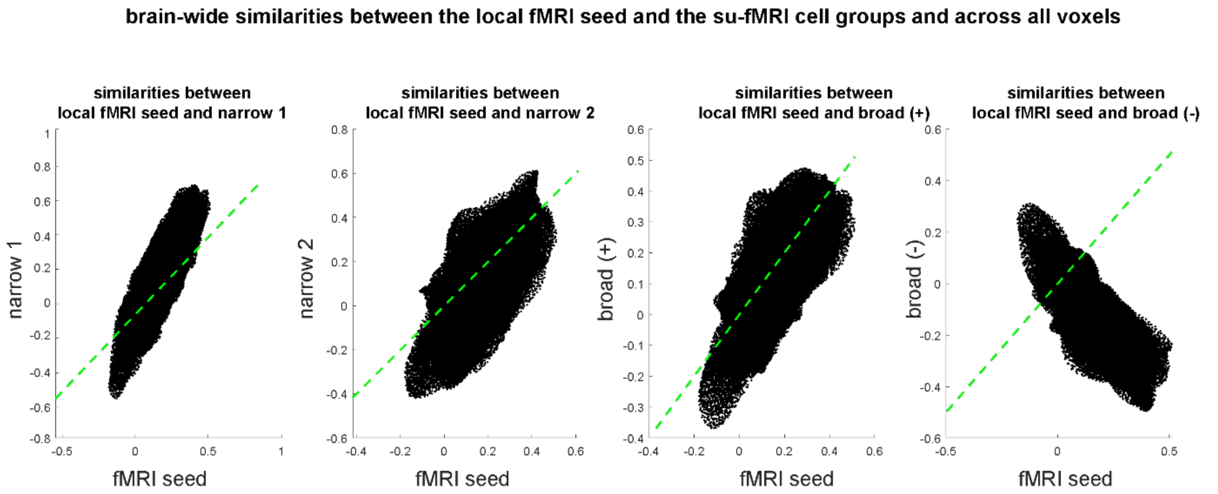

Figure S8. Voxel-wise similarity between the local fMRI seed map and each fMRI-cell group across the entire brain. Each panel shows a scatter plot comparing voxel-wise values from the fMRI seed to the corresponding voxel values from one cell-group map (narrow 1, narrow 2, broad (+), broad (–). Points represent all brain voxels, including both cortical and subcortical regions. Solid colored lines indicate the best-fit linear regression for each comparison, and the green dashed line denotes the identity line. Differences in slope and spread reflect how strongly each cell-group map aligns with the spatial pattern of the fMRI seed at the voxel level.

bioRxiv preprint doi: https://doi.org/10.64898/2026.06.25.734567; this version posted June 28, 2026. The copyright holder has placed this preprint (which was not certified by peer review) in the Public Domain. It is no longer restricted by copyright. Anyone can legally share, reuse, remix, or adapt this material for any purpose without crediting the original authors.

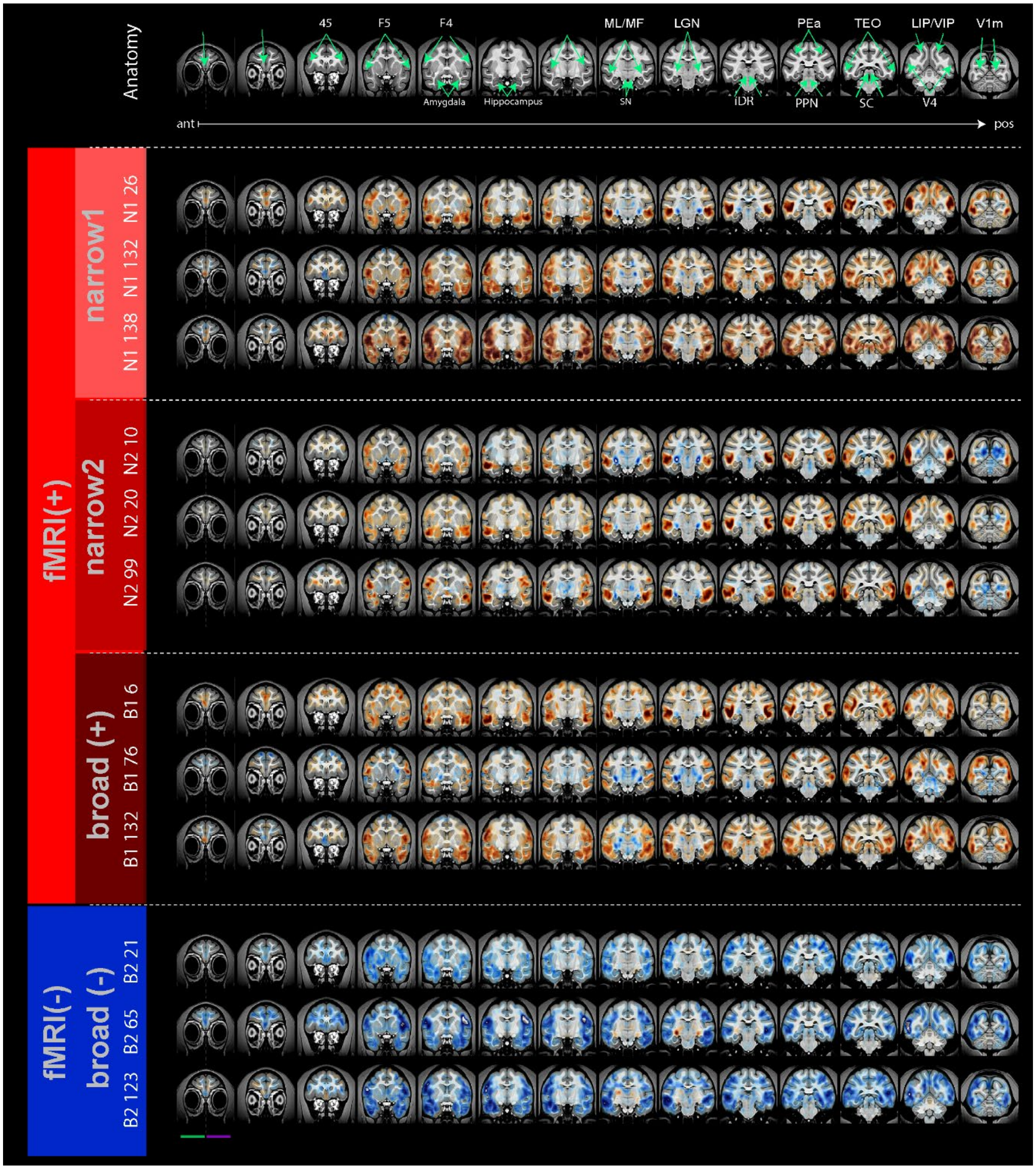

Figure S9. Example of single-unit fMRI maps. Each panel shows three representative fMRI maps derived from individual neurons, grouped according to their waveform-based classification (narrow 1, narrow 2, broad (+), broad (-). These examples illustrate the range and spatial patterns of whole-brain functional connectivity associated with single-unit activity within each neuronal subgroup.

bioRxiv preprint doi: https://doi.org/10.64898/2026.06.25.734567; this version posted June 28, 2026. The copyright holder has placed this preprint (which was not certified by peer review) in the Public Domain. It is no longer restricted by copyright. Anyone can legally share, reuse, remix, or adapt this material for any purpose without crediting the original authors.

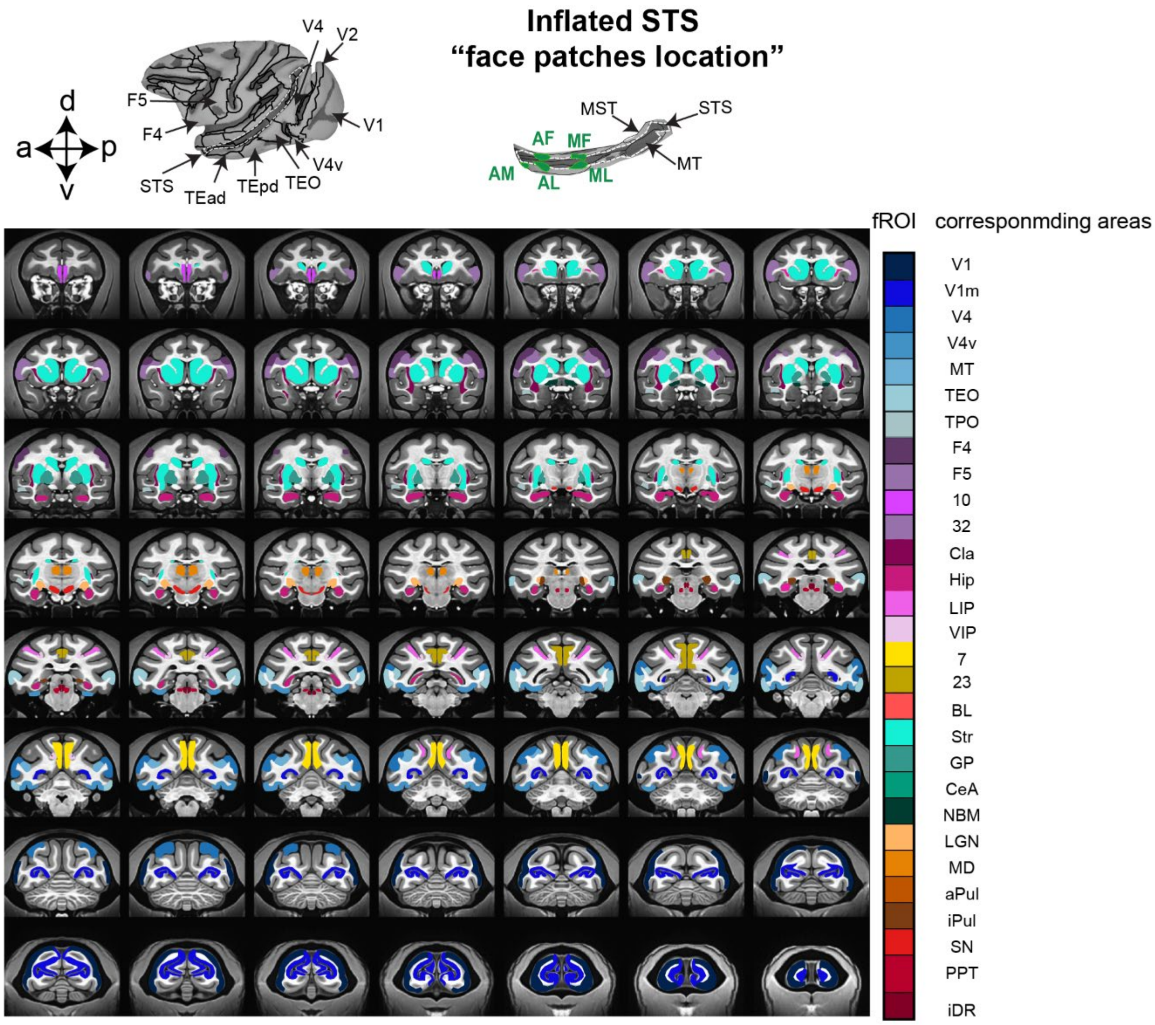

Figure S10. Functional regions of interest. Boundaries of the fROIS that were utilized in our current study.
